# Cyclic Electrodeposition of PtCu Alloy: Facile Fabrication of Highly Porous Platinum Electrodes

**DOI:** 10.1002/adma.201200806

**Published:** 2012-05-02

**Authors:** Arne Kloke, Christian Köhler, Ramona Gerwig, Roland Zengerle, Sven Kerzenmacher

**Affiliations:** Laboratory for MEMS Applications, IMTEK - Department of Microsystems Engineering, University of FreiburgGeorges-Koehler-Allee 103, 79110 Freiburg, GermanyE-mail: kerzenma@imtek.uni-freiburg.de; BIOSS - Centre for Biological Signalling Studies, Albert-Ludwigs-UniversitätFreiburg, Germany; Natural and Medical Sciences Institute at the University of TübingenMarkwiesenstrasse 55, 72770 Reutlingen, Germany

Porous platinum, bringing together high catalytic activity and a high catalyst surface area, is of high interest for a wide range of applications such as sensors, chemical engineering and fuel cells.^1^, ^2^ For instance in fuel cells, state of the art electrodes for electrooxidation of hydrogen, methanol, ethanol, formic acid, glucose as well as for reduction of oxygen consist of platinum or platinum-alloy catalysts.^1^, ^3^, ^4^

Several strategies exist for the fabrication of porous platinum electrodes ranging from the use of nanoparticles, over electrodeposition to dealloying.^2^ Nanoparticles have attracted great attention for catalysis because of their high surface to volume ratios (high platinum utilization). Depending on the diameter, catalytic effects have been observed for nanoparticles not known for the corresponding bulk material such as higher catalytic activity per surface area or preferential reaction pathways.^5^–^8^ For application as electrocatalyst the nanoparticles synthesized by, for example, wet chemical methods,^6^ need to be attached to a conductive support material and get connected to a charge collector (e.g., by spray coating) which requires additional process steps in electrode fabrication.^2^, ^4^ Moreover, weak nanoparticle-support or support-charge-collector interconnections can lead to a limited stability of such electrodes.^9^

Electrodeposition enables a facile fabrication of strongly adherent electrocatalysts on a conductive substrate within only one step.^2^ Techniques such as pulsed galvanostatic deposition (alternation of deposition and concentration relaxation) enable the generation of dendritic structures.^10^ Sacrificial templates such as microspheres,^11^ liquid crystals,^12^, ^13^ polymers^14^ or mesoporous structures (e.g., silica or alumina membranes)^15^–^17^ can be used to additionally control/design the pore structure on the corresponding scale (50 nm to 10 μm). Either way, so far electrodeposited platinum electrodes do not allow for roughness factors (RF, ratio of internal electrode surface to geometric area) higher than 1000, due to the fragile nature of the dendritic deposits.^10^, ^17^

The dealloying of a less noble metal (e.g., Cu, Ag, Al, Co) from platinum alloys represents a powerful alternative to the previously discussed techniques bringing together the advantage of a self-supporting structure, feature sizes in the nanometer range and high roughness factors of 3000 and higher.^2^, ^16^, ^18^–^22^ In general the alloy is either obtained as a bulk material by techniques such as arc-melting^19^ or the alloying partner is deposited onto a platinum layer and the resulting bilayer is subsequently annealed for alloy formation.^20^ Either way, the less noble alloying partner is subsequently removed by either chemical or electrochemical means leaving behind a porous platinum structure.^2^ Liu et al. showed an electrochemical way using a potentiostatic co-deposition of platinum and copper for alloy formation on glassy carbon followed by electrochemical dealloying of copper. In contrast to techniques such as arc melting this process can be performed at room temperature and in principle on every conductive substrate, making it highly compatible with other steps in device fabrication. Unfortunately, roughness factors of about only 700 have been achieved so far.^23^

Here we report a new cyclic electrodeposition procedure for facile fabrication of platinum-based electrodes with a roughness factor higher than 3000 based on multiple repetitions of electrochemical co-deposition of platinum-copper alloy and subsequent electrochemical dealloying of the less noble copper. We characterize the resulting electrodes by their surface roughness, microstructure and elemental composition (residual copper content) in dependence of the number of deposition cycles. Moreover, we demonstrate the advantages of the new process by application to implantable glucose fuel cells.

During fabrication the substrate (platinum evaporated on silicon wafer pieces, see Experimental Section) is subjected to cyclic voltammetry (CV) scans in sulfuric acid with additions of H_2_PtCl_6_ and CuSO_4_. Each deposition cycle consists out of a cathodic scan during which a layer of platinum-copper alloy is deposited and an anodic scan during which copper gets dissolved from the alloy leaving behind a porous platinum structure.

**Figure 1**A compares CV scans of a first deposition cycle performed on platinum substrates in H_2_SO_4_ containing either H_2_PtCl_6_ and CuSO_4_ (PtCu electrolyte) or H_2_PtCl_6_ only (Pt-only-electrolyte) to deposition in pure sulfuric acid. Here -0.25 V vs. SCE is chosen as negative potential to avoid superposition of the deposition processes by hydrogen generation currents. Comparing CV scans of the Pt-only electrolyte to pure H_2_SO_4_, additional reductive currents can be observed in the cathodic scan for potentials more negative than approximately 0.50 V vs. SCE (see label (1) in Figure 1A). This potential corresponds to the standard redox-potential for the reduction of [Pt(IV)CI_6_]^2^^−^ to metallic platinum:^24^


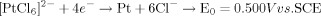
(1)

**Figure 1:**
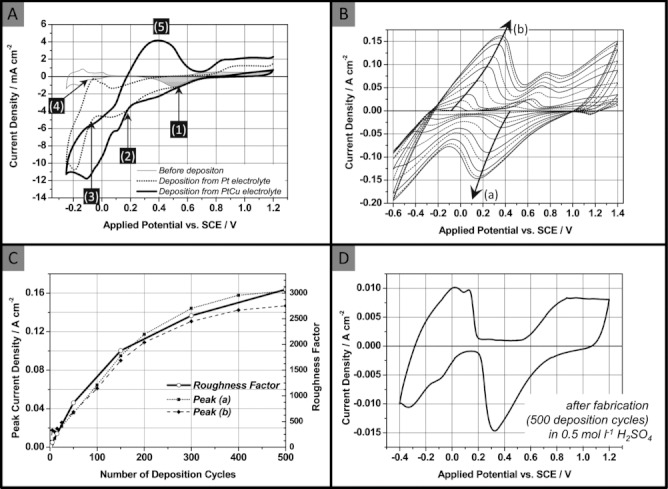
A) Comparison of CV scans (scan rate 50 mV s^−1^, between 1.20 and -0.25 V vs. SCE) performed on platinum substrates before deposition (in H_2_SO_4_, 0.5 m) and during first deposition cycle in Pt-Cu-electrolyte (in 0.5 m H_2_SO_4_ with H_2_PtCl_6_ and CuSO_4_, 0.02 m each). A first deposition cycle recorded in Pt-only-electrolyte (0.5 m H_2_SO_4_ with H_2_PtCl_6_) is additionally shown to visualize the effect of Cu-addition. B) Evolution of CVs (50 mV s^−1^, 1.40 to -0.60 V vs. SCE) during electrode fabrication in PtCu-electrolyte, represented by selected CV-cycles: 2, 10, 25, 50, 100, 150, 200, 300, 400 and 500. C) Evolution of peaks (a) and (b) from (B) in comparison to the evolution of roughness factors (subsequently recorded in 0.5 m H_2_SO_4_) observed at increasing number of deposition. D) CV scan recorded at 5 mV s^−1^ in 0.5 m H_2_SO_4_ of an electrode fabricated with 500 deposition cycles.

During the cathodic scan, there are two potentials at which an increase in deposition currents are observed (see labels (1) and (3)), indicating the beginning of a specific reduction process. This phenomenon is known in literature and was explained to originate from the presence of different Pt(IV) complexes resulting from hydrolysis of H_2_PtCl_6_ and accordingly different redox-potentials.^25^ On the subsequent anodic scan two peaks appear at -0.05 V and 0.01 V vs. SCE (see label (4)) reflecting the desorption of under potential deposited (UPD) hydrogen known from CVs of platinum in sulfuric acid. Reductive currents and thus platinum deposition are still observed during the anodic scan until approximately 0.45 V vs. SCE.

The addition of CuSO_4_ to the electrolyte results in increased reductive currents in the cathodic scan for potentials between 0.50 V and 0.25 V vs. SCE and for potentials more negative than 0.17 V. The additional reductive currents between 0.50 V and 0.25 V (between label (1) and (2)) are attributed to the UPD of copper, whereas bulk-deposition of copper and thus co-deposition of PtCu alloy is obtained for potentials more negative than 0.17 V vs. SCE (see label (2)).^26^ During the anodic scan an anodic current peak (see label (5)) is observed for potentials higher than 0.12 V vs.SCE reflecting the dissolution (dealloying) of copper from the just deposited alloy. As a consequence of the combined alloy deposition and dealloying process the electrode shows an about 2.2 times larger surface area after one deposition cycle in PtCu-electrolyte compared to before deposition.

To fabricate electrodes with a high surface roughness the previously discussed processes are multiply repeated. Here, the surface generated by the previous deposition cycle serves as substrate for the next cycle. Figure 1B shows selected curves of CV scans (between 1.20 V and -0.60 V vs SCE) observed during fabrication. Here, a potential of -0.60 V vs. SCE was chosen as negative scan limit, since this enables an increased surface area enlargement (factor 4.8) compared to -0.25 V vs. SCE (factor 2.2) during the first deposition cycle. Consequently, currents resulting from the generation of hydrogen gas appear in the CVs for potentials more negative than -0.25 V vs. SCE and overlap with the processes of platinum and copper deposition shown in Figure 1A.

For increasing number of deposition cycles a continuous increase of the peak current densities is observed in Figure 1B, reflecting the continuous growth of the electrode surface. In Figure 1C the corresponding peak current densities observed for peak (a) reflecting Pt−O reduction superimposed by deposition currents and peak (b) reflecting hydrogen oxidation (H_2_ and H-UPD oxidation) are shown in comparison to the roughness factors evaluated by CV scans in 0.5 m H_2_SO_4_ subsequent to fabrication. Their strong correlations to the roughness factor observed for the peak current densities of peak (a) and (b) make them powerful indicators for online monitoring of the surface growth. Figure 1D presents a CV recorded in H_2_SO_4_ of an electrode fabricated with 500 deposition cycles clearly showing the typical shape of platinum. According to the high surface roughness slow scan speeds of 5 mV s^−1^ and lower are required to resolve the typical shape of platinum and thus enable the measurement of the roughness factor.

**Figure 2** shows electron micrographs and **Table 1** summarizes physical properties of electrodes fabricated with a negative scan limit of -0.60 V vs. SCE and different numbers of deposition cycles. For electrodes fabricated with 500 deposition cycles in the PtCu-electrolyte a mean roughness factor of 3070 ± 300 is observed, which to our best knowledge is higher than elsewhere reported for electrodeposited platinum electrodes. The thickness of such an electrode structure amounts to 19 ± 4 μm. Without the presence of copper (Pt-only-electrolyte) the application of 500 deposition cycles result in an about three times lower roughness factor of 1040 ± 260 compared to in PtCu-electrolyte. This clearly demonstrates the effect of the combined alloy-deposition/dealloying process occurring in the PtCu-electrolyte on structure generation. When the process is performed on gold-covered instead of platinum-covered substrates very similar, stable structures are obtained. Here, the roughness factor after 500 deposition cycles amounts to 3410 ± 300, demonstrating the transferability of the deposition process to substrates other than platinum.

**Figure 2:**
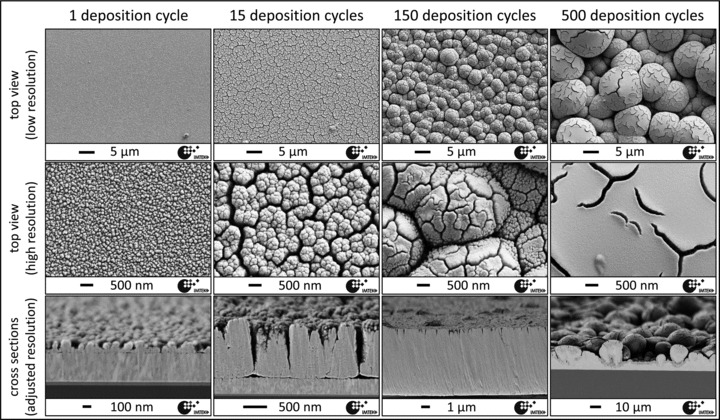
Scanning electron microscopy (SEM) images of electrodes fabricated with different number of deposition cycles.

**Table 1:** Physical properties. All electrodes were fabricated using the same deposition parameters but different numbers of deposition cycles

Number of Deposition Cycles	0	1	5	15	50	150	300	500	500_Pt
Roughness factor (RF)^a)^	4.4 ± 0.4	21.2 ± 1.1	92.0 ± 9.6	262 ± 41	864 ± 71	1880 ± 230	2560 ± 41	3070 ± 300	1040 ± 260
Average increase in RF per cycle	-	21.2	18.4	17.5	17.3	12.5	8.5	6.1	2.1
Specific surface area [cm^2^ mg^−1^]^b)^	-	n.e.	259 ± 251	241 ± 62	233 ± 23	184 ± 37	176 ± 18	162 ± 23	108 ± 33
Percentage of surface platinum atoms [%]^b)^	-	n.e.	11.0 ± 10.7	10.2 ± 2.6	9.9 ± 1.0	7.8 ± 1.6	7.4 ± 0.8	6.9 ± 1.0	4.6 ± 1.4
Cu-content (by EDX) [at%]^c)^	0	6 ± 1	18 ± 2	20 ± 2	21 ± 2	13 ± 1	14 ± 2	14 ± 2	n.e.
Cu-content (by XPS) [at%]^d)^		6 ± 2	n.e.	6 ± 1	n.e.	3 ± 1	n.e.	3 ± 1	n.e.
Layer thickness [μm]^e)^	0	0.10 ± 0.01	n.e.	1.05 ± 0.02	n. e.	6.6 ± 0.1	n.e.	19.1 ± 4.4	n.e.
Feature size [μm]^f)^	-	0.07 ± 0.01	0.19 ± 0.03	0.45 ± 0.07	1.02 ± 0.25	3.5 ± 0.9	7.0 ± 2.9	8.8 ± 5.3	-

n.e. not evaluated; ^a)^calculated from 4-8 samples each, error is given as standard deviation; ^b)^calculated from at 4-8 samples each (see Experimental Section), error is calculated by Gaussian error propagation of standard deviations of deposited mass and roughness factor; ^c)^calculated from EDX-analysis of two samples; ^d)^average of two samples each, error value represents the difference between sample values and average value; ^e)^calculated from SEM images of cross sections prepared by cross section polisher, error represents standard deviation of three to nine analyzed images of a representative sample; ^f)^feature size has been determined from top-view SEM images as shown in supplementary material, error represents standard deviation between at least 9 feature diameters recorded for two samples.

From data shown in Table 1 a saturation of the roughness factor with increasing number of deposition cycles is observed in spite of a continuously increasing substrate area for each deposition cycle. This saturation can clearly be seen from values listed for average increase in roughness factor per deposition cycle. It can be attributed to two main effects that decelerate the growth process: On the one hand, deposition leads to a depletion of platinum and copper ions in the electrolyte solution. This depletion effect is even more prominent inside the growing porous regions within the electrode structure due to longer diffusion pathways. On the other hand deposition inside of pores leads to pore closure. Accordingly, the deeper layers of the electrodes appear to be of a bulky, non-porous nature in the cross-section images shown in Figure 2. This pore closure can explain the decreasing percentage of surface to bulk platinum atoms (see Experimental Section for details) from 11.0% to 6.9% which is observed for increasing the number of deposition cycles from 5 to 500.

As can be seen in Figure 2, the top layers of the electrodeposited structure are of a more porous nature and consist out of spherical building blocks. The mean diameter of these building blocks is listed as feature size in Table 2 and shows a similar increase with number of deposition cycles as the surface roughness. The coarsening of the spherical building blocks observed with increasing deposition cycles, suggests an agglomeration or fusion process of neighboring building blocks during the growth and deposition process.

The bulky nature of the electrode structure in the deeper regions leads to a lower platinum mass utilization (162 cm^2^ mg^−1^ in case of 500 deposition cycles) compared to e.g. the method of Liu (322 cm^2^ mg^−1^)^23^ or templated fabrication techniques (470 cm^2^ mg^−1^)^27^ on the one hand. But on the other hand it enables good adhesion and mechanical stability of the electrode structure. After 5 min in an ultrasonic bath (240 W, 35 kHz) three out of three electrodes showed a difference in roughness factor of 7% at most. This is not substantial regarding the precision of the measurement method, in particular when considering possible misalignment during reassembly of the electrodes after ultrasonic treatment.

Energy dispersive X-ray spectroscopy (EDX) and X-ray photoelectron spectroscopy (XPS) analysis showed that copper is still present in the deposited structure despite of dealloying periods within each cycle (see Table 1). Comparison of XPS data (penetration depth of a few nm) to EDX data (penetration depth of a few hundred nm) reveals a significantly lower copper content at the surface compared to in the bulk. These finding indicate the electrode structure to consist out of a copper rich bulk and a copper-poor platinum shell. Such a PtCu-bulk/Pt-shell configuration has previously been described for PtCu dealloying systems, e.g. by Strasser and coworkers.^28^ Incomplete dissolution of the less noble alloying partner is a common observation in dealloyed structures. Dealloying is known to be a rearrangement process, which is kinetically limited by the surface diffusivity of the noble metal component.^2^, ^29^, ^30^ If the rearrangement process occurs too slowly a passivation layer forms, which is characterized by a fraction of the less noble metal below the parting limit and no further dissolution of the less noble metal from the bulk takes place. Moreover, the enclosure of copper during cyclic electrodeposition is promoted by three properties of the process (see Figure 1): first, negative current densities are observed during the anodic scan in the copper dissolution range (>0.10 V vs. SCE) in the Pt-only-electrolyte indicating an ongoing platinum deposition. Second, the dealloying time given during the anodic CV scan is limited baring the risk that copper is not dissolved completely. Third, during the subsequent cathodic scan first pure platinum is deposited onto the just generated porous layer before co-deposition takes place.

An application example in which a high surface roughness plays a crucial role is the implantable glucose fuel cell based on platinum catalysts. This type of fuel cell is currently being developped as a sustainable alternative to conventional batteries for the supply of low power medical implants.^31^–^33^ Here, electricity is generated by the electrooxidation of glucose and electroreduction of dissolved oxygen from body fluids at two spatially separated electrodes. Different to other fuel cell systems, anode and cathode are operated in the same media. Since platinum is catalytically active for both electrode reactions, mixed potentials occur due to the simultaneous presence of both reactants, reducing the achievable cell voltage. At the anode, the use of highly porous platinum electrodes has recently been shown to minimize the influence of oxygen on the anode potential.^34^, ^35^

In **Figure 3**A open circuit electrode potentials are shown for electrodes fabricated with different number of deposition cycles. For increasing number of deposition cycles and thus increasing surface roughness, a trend towards more negative electrode potentials is observed. At 7% oxygen saturation (estimated upper physiological concentration in tissue^36^), electrode potentials are observed for up to five deposition cycles which correspond to typical cathode potentials obtained in implantable glucose fuel cells. Typical anode potentials are observed for electrodes fabricated with fifty deposition cycles and more.

**Figure 3:**
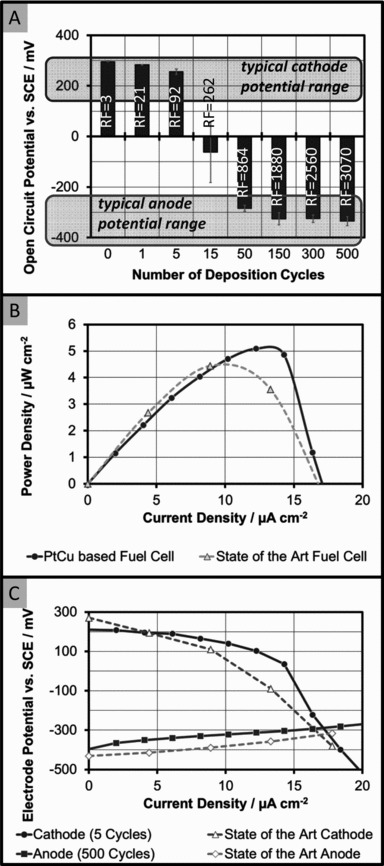
Application of electrodes fabricated by cyclic electrodeposition of PtCu-alloy to potentially implantable glucose fuel cells. All data has been recorded at 7% oxygen saturation and 3 mm glucose in phosphate buffered saline (estimated physiological concentrations^36^) at 37 °C. A) Open circuit potentials of electrodes fabricated with different number of deposition cycles (data is recorded after 3 h @ 7% oxygen saturation, average values of 2 samples each). B,C) Load curve comparison of glucose fuel cells using electrodes fabricated by cyclic electrodeposition (average value of six fuel cells) to state of the art fuel cells (data from 35, average of three fuel cells). B) shows power densities and (C) shows the corresponding electrode potentials.

The dominance of glucose oxidation potential on the electrode potential at high surface roughness is a consequence of the about 100 times lower concentration of oxygen compared to glucose at physiological conditions:^36^ Whereas the cathodic oxygen reduction is diffusion controlled at the low oxygen concentration under physiological conditions, the glucose oxidation is kinetically controlled and thus can profit from an increase in surface area, promoting the anodic character of the mixed potential for electrodes fabricated with fifty deposition cycles and more.^17^, ^37^

A complete glucose fuel cell using cyclic electrodeposition of PtCu alloy was assembled from anodes fabricated with 500 deposition cycles and cathodes fabricated with 5 deposition cycles. Slitted silicon chips with evaporated Pt layer were used as cathode substrates (see 35 for details). With a maximum of 5.1 μW cm^−2^ (average of six fuel cells, see Figure 3B) these fuel cells exhibit a power density of even 16% more than reported for state of the art fuel cells (4.4 μW cm^−2^).^35^ This enhanced power density is mostly related to improved mass transport properties of the cathode compared to state of the art (see Figure 3C). State of the art fuel cells use cathodes and anodes that differ in material as well as in fabrication process. In contrast to this, the reaction-specificity of the electrodes fabricated according to the present work is achieved by only adjusting the number of deposition cycles.

In conclusion we demonstrated a novel electrodeposition process for the fabrication of highly porous platinum-based electrodes based on alternation of co-deposition and dealloying by cyclic voltammetry at room temperature in a simple acidic electrolyte containing H_2_PtCl_6_ and CuSO_4_. This process enables the generation of platinum electrodes with a surface-near residual copper content of 3 ± 1 at% (determined by XPS-analysis) and a roughness factor of more than 3000. The resulting surface roughness can be tuned by choosing the appropriate number of deposition cycles. Online process control is enabled by using the evolution of the peak current densities in the cyclic voltammograms as online-indicator for the growing electrode surface area. The novel process is a facilitated and more variable method compared to state of the art: no elevated temperatures, templates or surfactants are required. Moreover, the here presented process is in principle not limited to a specific substrate, but may be applied on every conductive substrate which here has already been demonstrated on platinum and gold surfaces. Since only 6.9% to 11.0% of the platinum is available at the catalyst surface the presented process is not well-suited to large area applications. Nevertheless, this process is relevant for small scale and niche applications such as (micro) fuel cells, electrochemical sensors or stimulation electrodes in which material properties are more important than material costs.^37^–^39^

## Experimental Section

*Electrode fabrication*: Substrates are fabricated by evaporation of 50 nm of titanium and 250 nm of platinum onto a 4’’ silicon wafer (<100> orientation, 525 μm thickness, n^+^ type doped). Before use samples were cleaned in acetone, isopropyl alcohol and water in an ultrasonic bath and weighted (SC 2, Sartorius, Göttingen, Germany). A screwed assembly of polycarbonate and silicone rubber pieces was used to connect two platinum wires (50 μm diameter, Chempur, Karlsruhe, Germany) to the electrode, exposing a geometric area of 1 cm^2^ as shown in 40. Electrodes used in fuel cell assemblies were fabricated with a geometric area of 2.25 cm^2^. Ten CV scans (50 mV s^−1^, 1.40 to −0.40 V vs. SCE) in 0.5 m H_2_SO_4_ (Merck, Darmstadt, Germany) were performed under N_2_ atmosphere to electrochemically clean the substrate's surface. During fabrication and characterization a potentiostat (PCI4/300, Gamry Instruments, Warminster PA, USA) was used with a platinum counter electrode and a saturated calomel reference electrode (SCE, KE11, Sensortechnik Meinsberg, Ziegra-Knobelsdorf, Germany).

*Electrode characterization*: Subsequent to fabrication CV scans in H_2_SO_4_ (0.5 m) were performed to clean the electrodes (10 cycles, 50 mV s^−1^, 1.40 to -0.40 V vs. SCE). Additional scans with adjusted negative scan limit and scan rate were applied to electrochemically determine the surface area according to 41. Electron micrographs of all samples were taken with a Zeiss Supra 60 VP. EDX data were recorded using a Thermo SytemSix (analysis area: 2 × 2 mm^2^). Cross sections were generated using the cross section polisher Jeol SM-09010. XPS data were recorded by ten sweeps from 0 to 1360 eV over an analysis area of 1.4 × 1.4 mm^2^ using a high resolution XPS-instrument PHI Quantera SXM (X−ray source: Al Kα E = 1486.6 eV). Deposited masses were determined by comparison of sample masses before and after deposition under consideration of the copper content in the electrode determined by EDX. Based on the electrochemically determined surface area *A*_echem_ and the deposited masses *m*_dep_, the percentage of surface atoms *r*_sa_ was calculated according to:


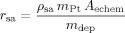
(2)

Here *ρ*_sa_ is the average density of surface platinum atoms (1.31 10^15^ atoms cm^−2^)^41^ and *m*_Pt_ the mass of a platinum atom (3.24 10^−19^ mg).

*Application to glucose fuel cells*: Different to the other electrodes, cathodes for fuel cell tests were fabricated on permeable, slitted silicon substrates as described in a previous publication.^35^ As shown there, fuel cells were assembled with this permeable cathode mounted in front of the anode.^35^ An additional filter membrane (Supor-450, Pall Life Sciences, East Hills NY, USA) was set on top of the fuel cell to simulate the transport resistance expected to occur due to tissue encapsulation after device implantation.^35^

Subsequent to assembly, electrodes and complete fuel cells, respectively, were autoclaved in an aseptic electrochemical reactor.^40^ All experiments were conducted at 37 °C in freshly prepared phosphate buffered saline (PBS, PBS tabs, pH = 7.4, 0.143 m chloride ions, Invitrogen, Karlsruhe, Germany) with an addition of α−D(+) glucose monohydrate (3 mm, Carl-Roth, Karlsruhe, Germany) and an oxygen saturation of 7% according to physiological concentrations in human tissue fluids.^36^ For the construction of polarization curves galvanostatic loads was successively increased by 2 μA cm^−2^ from 0 to 20 μA cm^−2^ using our parallel electrochemical testing environment described in 40. Galvanostatic loads were increased every 6 h, the last value of each load step was used to finally construct polarization curves.
